# Supplementary material for: Evolution of Malaria Incidence in Five Health Districts, in the Context of the Scaling up of Seasonal Malaria Chemoprevention, 2016 to 2018, in Mali
Source: Int J Environ Res Public Health. 2021 Jan 19;18(2):840. doi: 10.3390/ijerph18020840 (PMC7844620; doi:10.3390/ijerph18020840)
Supplement: Supplementary file 1 [file ijerph-18-00840-s001.pdf]

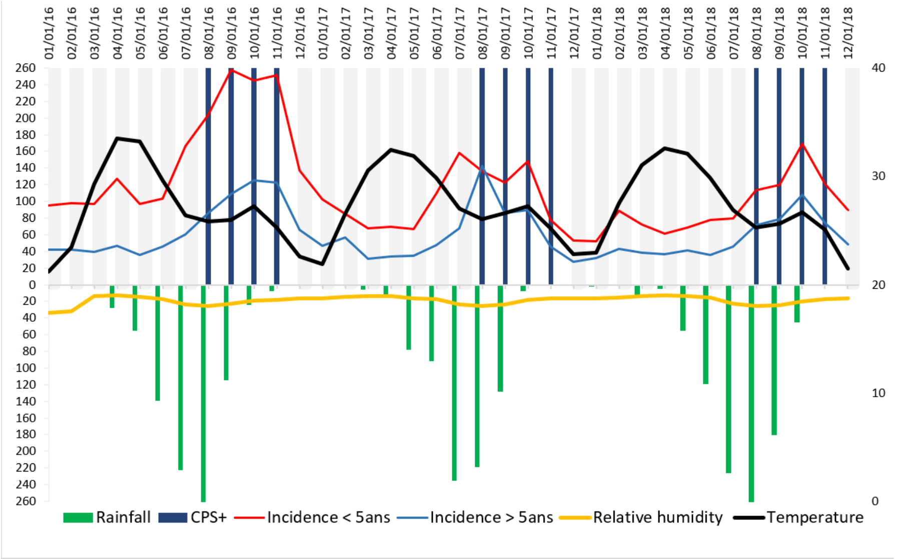

Figure S1: Meteorological variables and incidence of malaria by age group and dates of implementation of Seasonal Malaria Chemoprevention (SMC) in Kadiolo health district.

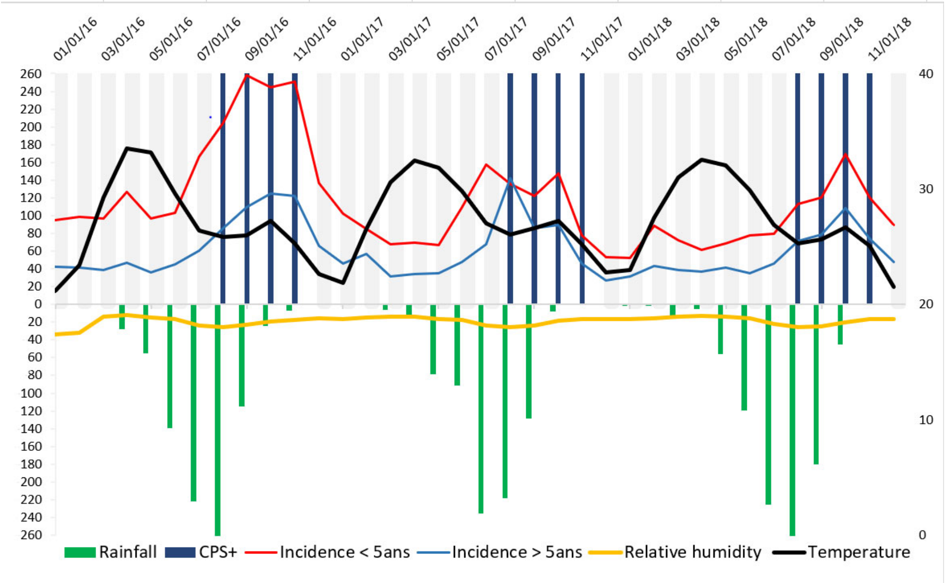

Figure S2: Meteorological variables and incidence of malaria by age group and dates of implementation of Seasonal Malaria Chemoprevention (SMC) in Kati health district.

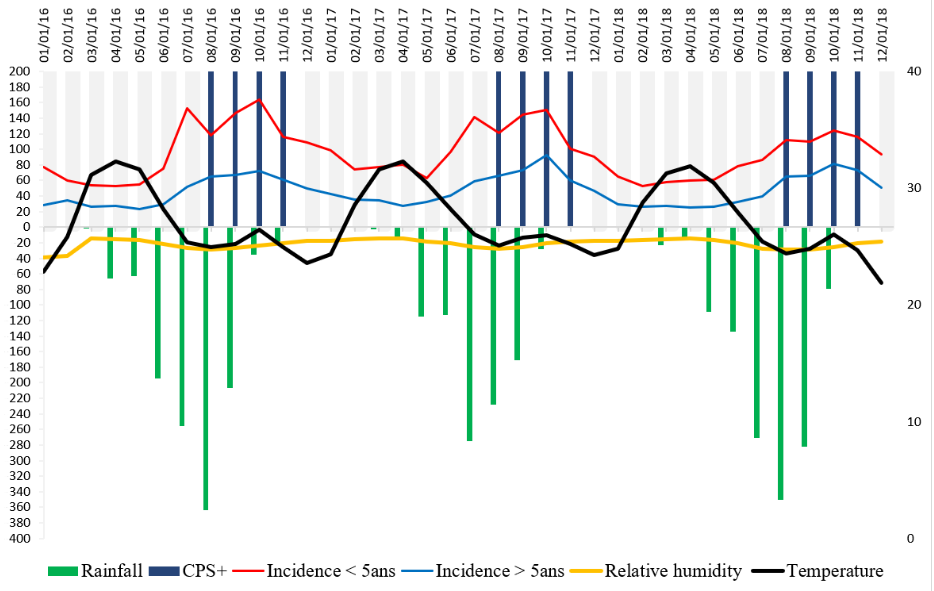

Figure S3: Meteorological variables and incidence of malaria by age group and dates of implementation of Seasonal Malaria Chemoprevention (SMC) in Sikasso health district.

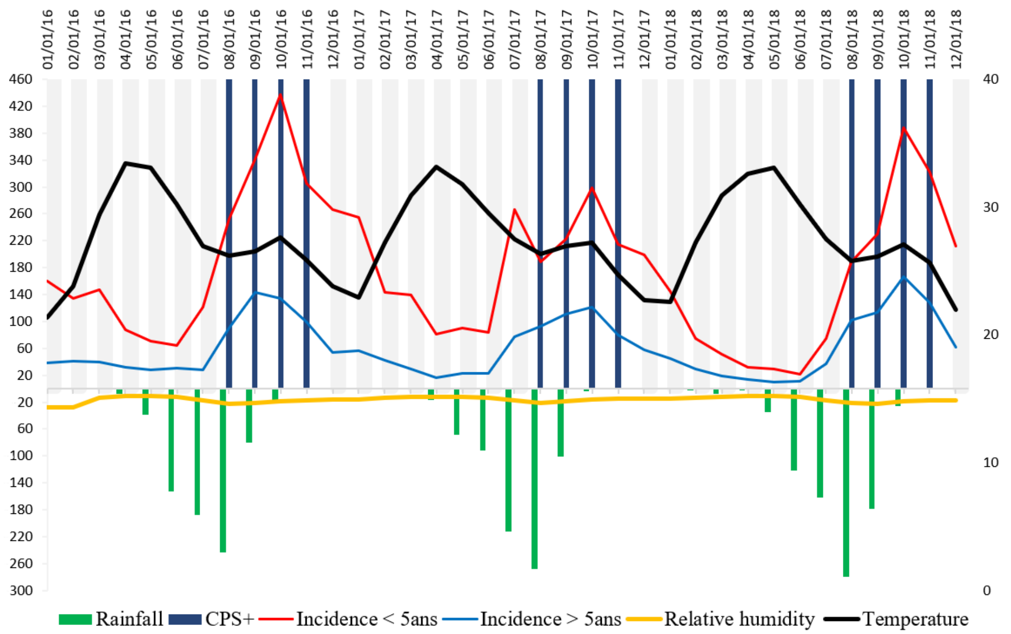

Figure S4: Meteorological variables and incidence of malaria by age group and dates of implementation of Seasonal Malaria Chemoprevention (SMC) in Touminian health district.

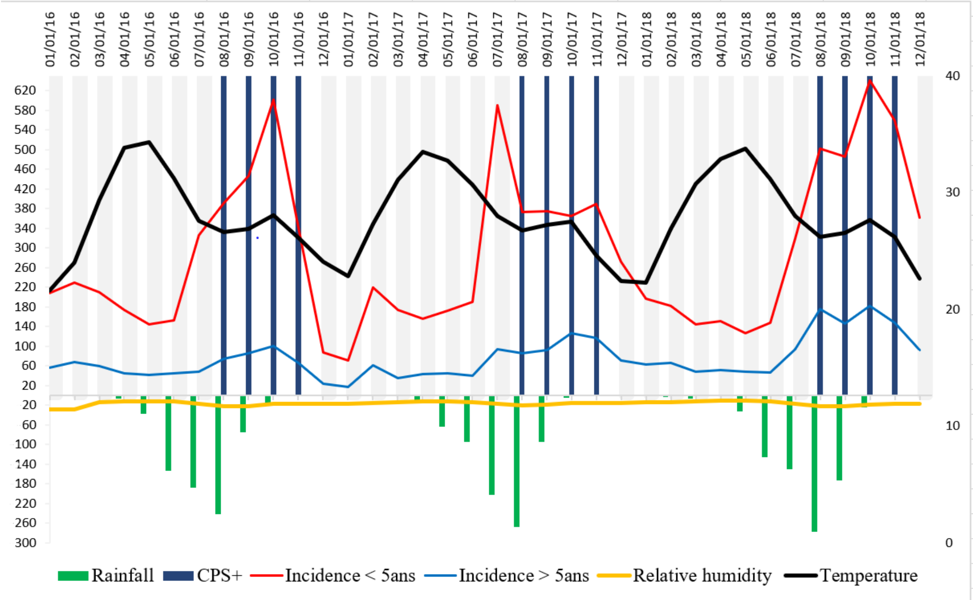

Figure S5: Meteorological variables and incidence of malaria by age group and dates of implementation of Seasonal Malaria Chemoprevention (SMC) in Yorosso health district.

Comments:

Meteorological variables and malaria incidence by age group and dates of implementation of Seasonal Malaria Chemoprevention (SMC).

At the top, the red curve represents the malaria incidence of children under 5 years of age and the light blue curve represents the malaria incidence of children over 5 years of age; the dark blue histogram represents the months of CPS implementation. The black curve represents the median temperature projected on the secondary axis.

At the bottom is the rainfall and relative humidity.

The figures show that seasonal malaria chemoprevention (SMC) was always implemented after the start of the high transmission period in all health districts.
